# Baseball injuries in adolescent athletes with ADHD

**DOI:** 10.3389/fspor.2022.1032558

**Published:** 2023-01-09

**Authors:** John M. Feldkamp, Adam R. Stevens, Syler R. Blaakman, Elijah W. Hale

**Affiliations:** ^1^ University of Rochester, Rochester, United States; ^2^ School of Medicine, University of Colorado Anschutz Medical Campus, Aurora, United States

**Keywords:** ADHD, baseball, stimulants, controversy, sports

## Abstract

At the intersection of injury-prone sports such as baseball and conditions like ADHD that affect all aspects of life, there is a lack of research. This limits the availability of preventive care programs designed to target potential risks and promote a safe experience. In this retrospective cohort study, we assess the frequency of injury in youth baseball players with and without ADHD, along with further investigation into how treatment with stimulant medication may modify risk factors. The data for this study were obtained in deidentified, aggregate format from the TriNetX research database. We identified all patients under 25 years of age with a designation of baseball activity. Within this population, we separated patients by presence or absence of ADHD diagnosis, and then by stimulant usage. The studied outcomes were injuries commonly occurring in baseball, including fractures, sprains, and specific injury patterns. We identified 17,710 patients under 25 years old with designated baseball activity, 1,183 of which had a diagnosis of ADHD. Of these, 511 had a history of stimulant medication and 470 had no history of stimulant use. For most events (i.e., injuries), there were no statistical differences between cohorts. The overall ADHD cohort significantly differed from the Not ADHD cohort in 3 events: “thorax, abdomen, pelvis injuries,” “ankle sprain,” and “concussion.” When athletes with ADHD received treatment, this trend reversed for select injuries: “any fracture”, “head or neck injuries”, “upper limb injuries”, and “lower limb injuries” were less likely in ADHD athletes on stimulants. Given the ongoing debate around stimulant use in athletics, our study is relevant to many patients, providers, and the baseball community.

## Introduction

Attention-deficit/hyperactivity disorder (ADHD) is one of the most common childhood neurodevelopmental disorders, affecting 6%–9% of children and adolescents in the United States (1). It is characterized by excessive hyperactivity, impulsivity, mood swings, and difficulty maintaining attention (2). ADHD often leads to difficulty at school and work, impaired relationships, low self-esteem, and risky behavior in organized activity, such as a sports (3). Organized sports, however, have also been shown to reduce symptoms in ADHD youth, often in team sports like baseball, particularly as hyperactive symptoms are known to be reduced after exercise (4).

Baseball is a longstanding facet of American culture. In 2020 alone, 3,403,000 children between the ages of 6–12 and 1,845,000 youths aged 13–17 played baseball; numbers which were even higher before the COVID-19 epidemic (5). However, the injury rate in baseball is higher than some other team sports (6). Similarly, ADHD is associated with far higher rates of injuries in all activities, not only sports (7). While hyperactive symptoms are improved by exercise, ADHD is known to impair motor function, reflexes, and situational awareness, all of which are important for avoiding injury in fast-paced sports like baseball (2, 8). Unfortunately, at the intersection of injury-prone sports such as baseball and conditions like ADHD that affect all aspects of life, there is a lack of research. This limits the availability of preventive care programs designed to target potential risks and promote a safe experience. In this retrospective cohort study, we assess the frequency of injury in youth baseball players with and without ADHD, along with further investigation into how treatment with stimulant medication is associated with particular risk factors. We hypothesize that our research will reinforce findings that individuals with ADHD will have higher rates of injury compared to players without ADHD. We also hypothesize that stimulant medications, known to effectively treat ADHD symptoms, may be associated with lower rates of injury in baseball players with ADHD compared to players without ADHD.

## Methods

The data for this study were obtained in deidentified, aggregate format from the TriNetX research database. TriNetX contains electronic medical records from large healthcare organizations (HCOs) and has been well-established for medical research, including a wide range of fields such as obstetrics (9), sexuality (10), and surgery (11). TriNetX, LLC is compliant with the Health Insurance Portability and Accountability Act (HIPAA), the US federal law which protects the privacy and security of healthcare data, and any additional data privacy regulations applicable to the contributing HCO (12). TriNetX is certified to the ISO 27001:2013 standard and maintains an Information Security Management System (ISMS) to ensure the protection of the healthcare data it has access to and to meet the requirements of the HIPAA Security Rule. Any data displayed on the TriNetX Platform in aggregate form, or any patient level data provided in a data set generated by the TriNetX Platform only contains de-identified data as per the de-identification standard defined in Section §164.514(a) of the HIPAA Privacy Rule (12).

Using International Classification of Diseases (ICD) codes, we identified all patients under 25 years of age with a designation of baseball activity (Y93.64) between August 11, 2002 and August 11, 2022. Within this population, we separated patients by presence or absence of ADHD diagnosis (F90). The ADHD cohort was further separated by stimulant usage. Stimulant use was defined by the presence of either amphetamine-class medications or phenidate-class medications individually. Patients with both amphetamine- and phenidate-class medications present at the same instance in their chart were excluded from analysis, as combined stimulant use is reserved for significantly severe cases (13).

Using the TriNetX software, we performed statistical analysis between the cohort without ADHD and each of the other groups, for a total of 3 analyses (Not ADHD vs. ADHD with stimulant use; ADHD without stimulant use; and ADHD overall). Prior to comparison, the cohorts were balanced based on age, sex, and race using nearest-neighbor matching to a difference in propensity scores <0.1 (10). After matching, cohorts had no significant differences in sex, age, or race. Table 1 contains cohort demographic information for the Not ADHD and overall ADHD cohorts, both before and after balancing on these characteristics. Balancing was performed before each of the three analyses. As the reference group without ADHD was matched to each of the above groups before analysis, the N of the reference group was equal to the matched group being analyzed.

**Table 1:** Demographics of overall ADHD cohort and not ADHD reference cohort, before and after propensity score matching on sex, race, and age.

Demographic	N	% of Cohort	*P*-value	Demographic	N	% of Cohort	*P*-value
(Pre-Matching)				(Post-Matching)			
**Current Age**		(Mean +/− SD)		**Current age**		(Mean +/− SD)	
Not ADHD	16,527	18.1 ± 4.12	<0.001*	Not ADHD	1183	17.7 ± 3.94	1
Overall ADHD	1183	17.7 ± 3.93		Overall ADHD	1183	17.7 ± 3.93	
**Male**				**Male**			
Not ADHD	10,335	63%	<0.001*	Not ADHD	858	73%	1
Overall ADHD	858	73%		Overall ADHD	858	73%	
**Female**				**Female**			
Not ADHD	5962	37%	<0.001*	Not ADHD	320	27%	1
Overall ADHD	320	27%		Overall ADHD	320	27%	
**White**				**White**			
Not ADHD	12,660	78%	0.177	Not ADHD	935	79%	1
Overall ADHD	935	79%		Overall ADHD	935	79%	
**Black**				**Black**			
Not ADHD	1089	7%	0.031*	Not ADHD	98	8%	1
Overall ADHD	98	8%		Overall ADHD	98	8%	
**Hispanic/Latino**				**Hispanic/Latino**			
Not ADHD	997	6%	0.366	Not ADHD	58	5%	0.007*
Overall ADHD	90	8%		Overall ADHD	90	8%	
**Not Hispanic/Latino**				**Not Hispanic/Latino**			
Not ADHD	10,269	63%	<0.001*	Not ADHD	774	66%	<0.001*
Overall ADHD	902	77%		Overall ADHD	902	77%	
**Unknown race**				**Unknown race**			
Not ADHD	2147	13%	0.015*	Not ADHD	126	11%	1
Overall ADHD	126	11%		Overall ADHD	126	11%	
**Unknown ethnicity**				**Unknown ethnicity**			
Not ADHD	5032	31%	<0.001*	Not ADHD	346	29%	<0.001*
Overall ADHD	186	16%		Overall ADHD	186	16%	

Asterisked values indicate *P* < 0.05.

The studied outcomes were injuries commonly occurring in baseball: “upper limb fractures,” consisting of fractures of the shoulder, humerus, forearm, wrist, or hand (ICD-10: S42, S52, S62); “lower limb fractures,” consisting of fractures of the femur, lower leg, ankle, or foot (ICD: S72, S82, S92); “any fractures,” consisting of any prior mentioned fracture type; “head & neck injuries” consisting of injuries to the musculoskeletal system above the clavicle (ICD: S00–19); “upper limb injuries” consisting of injuries from the shoulder to fingers (ICD: S40–69); “lower limb injuries” consisting of injuries from the hip to toes (ICD: S70–99); “thorax, abdomen, pelvis injuries” consisting of injuries from the clavicle to the hip, including the innominate and genitals (ICD: S40–69); shoulder sprain (ICD: S43.4); rotator cuff and/or labrum injury (ICD: M75.1, S43.42–43, S46.0); elbow sprain (ICD: S53.4); ulnar collateral ligament (UCL) damage (IDC: S53.44 & S53.3); knee sprain (ICD: S83.4–5); ankle sprain (S93.4); and concussion (ICD: S06.0). Injuries were considered binary variables based on presence or absence of the corresponding ICD code in the health record within the time window. A *t*-test was used to compare event rates between cohorts. Odds ratios with a 95% confidence interval were also calculated from event rates. The results are compiled in Table 2. Significance for this study was set at *P* < 0.05. As this study contained only deidentified aggregate data, the Colorado Multiple Institutional Review Board (COMIRB) designated it as non-human research not in need of approval.

**Table 2:** Cohort size, absolute risk, odds ratio with confidence interval, and *t*-test *P*-value by outcome. Stimulants abbreviated as “Stims.” Asterisked values indicate *P* < 0.05.

Event ICD 10	Cohort N	Event N	Risk	Odds Ratio	95% CI	*P*-value	Event ICD 10	Cohort N	Event N	Risk	Odds Ratio	95% CI	*P*-value
**Upper limb fractures**							**Shoulder sprains**						
Not ADHD	Reference cohort						Not ADHD	Reference cohort					
Overall ADHD	1183	239	20.2%	1.043	(0.85, 1.28)	0.68	Overall ADHD	1,183	37	3.1%	1.559	(0.93, 2.62)	0.092
ADHD−Stims	470	91	19.4%	1.07	(0.77, 1.49)	0.677	ADHD−Stims	470	10	2.1%	1.00	(0.41, 2.43)	1
ADHD + Stims	511	79	15.5%	0.742	(0.54, 1.03)	0.071	ADHD + Stims	511	13	2.5%	1.308	(0.57, 3.01)	0.527
**Lower limb fractures**							**Elbow sprains**						
Not ADHD	Reference cohort						Not ADHD	Reference cohort					
Overall ADHD	1183	91	7.7%	1.134	(0.83, 1.55)	0.428	Overall ADHD	1,183	32	2.7%	0.816	(0.51, 1.31)	0.399
ADHD−Stims	470	42	8.9%	1.55	(0.94, 2.55)	0.082	ADHD−Stims	470	10	2.1%	0.66	(0.29, 1.48)	0.311
ADHD + Stims	511	30	5.9%	1.118	(0.66, 1.91)	0.683	ADHD + Stims	511	14	2.7%	1.079	(0.50, 2.32)	0.845
**Any fractures**							**Knee sprains**						
Not ADHD	Reference cohort						Not ADHD	Reference cohort					
Overall ADHD	1183	374	31.6%	0.977	(0.82, 1.16)	0.791	Overall ADHD	1,183	30	2.5%	1.114	(0.66, 1.89)	0.688
ADHD−Stims	470	149	31.7%	1.07	(0.81, 1.41)	0.621	ADHD−Stims	470	13	2.8%	0.86	(0.41, 1.83)	0.701
ADHD + Stims	**511**	**127**	**24**.**9%**	**0**.**726**	**(0.55, 0.95)**	**0**.022*	ADHD + Stims	511	10	2.0%	0.765	(0.33, 1.76)	0.527
**Head or neck injuries**							**Ankle sprains**						
Not ADHD	Reference cohort						Not ADHD	Reference cohort					
Overall ADHD	1183	487	41.2%	1.160	(0.98, 1.37)	0.077	**Overall ADHD**	**1,183**	**138**	**11**.**7%**	**1**.**400**	**(1.07, 1.83)**	**0**.014*
ADHD−Stims	470	211	44.9%	1.23	(0.95, 1.60)	0.113	**ADHD−Stims**	**470**	**63**	**13**.**4%**	**1**.**92**	**(1.25, 2.97)**	**0**.003*
**ADHD + Stims**	**511**	**140**	**27**.**4%**	**0**.**563**	**(0.43, 0.73)**	**0**.001*	ADHD + Stims	511	36	7.0%	0.943	(0.59, 1.51)	0.809
**Upper limb injuries**							**Rotator cuff or labrum injury**						
Not ADHD	Reference cohort						Not ADHD	Reference cohort					
Overall ADHD	1,183	538	45.5%	1.063	(0.90, 1.25)	0.457	Overall ADHD	1,183	29	2.5%	1.461	(0.82, 2.60)	0.194
ADHD−Stims	470	213	45.3%	1.05	(0.81, 1.36)	0.694	ADHD−Stims	470	10	2.1%	1.00	(0.41, 2.43)	1
**ADHD + Stims**	**511**	**179**	**35**.**0%**	**0**.**691**	**(0.54, 0.89)**	**0**.004*	ADHD + Stims	511	11	2.2%	1.102	(0.46, 2.62)	0.825
**Lower limb injuries**							**UCL injury**						
Not ADHD	Reference cohort						Not ADHD	Reference cohort					
Overall ADHD	1,183	414	35.0%	1.147	(0.97, 1.36)	0.117	Overall ADHD	1,183	18	1.5%	0.716	(0.39, 1.32)	0.281
ADHD−Stims	470	166	35.3%	1.10	(0.84, 1.44)	0.492	ADHD−Stims	470	10	2.1%	0.91	(0.38, 2.16)	0.825
**ADHD + Stims**	**511**	**135**	**26**.**4%**	**0**.**760**	**(0.58, 0.99)**	**0**.046*	ADHD + Stims	511	12	2.3%	1.205	(0.52, 2.81)	0.666
**Thorax, abdomen, pelvis injuries**							**Concussion**						
Not ADHD	Reference cohort						Not ADHD	Reference cohort					
**Overall ADHD**	**1,183**	**150**	**12**.**7%**	**1**.**556**	**(1.19, 2.03)**	**0**.001*	**Overall ADHD**	**1,183**	**180**	**15**.**2%**	**1**.**862**	**(1.44, 2.41)**	**0**.001*
ADHD−Stims	470	49	10.4%	1.07	(0.70, 1.64)	0.745	**ADHD−Stims**	**470**	**74**	**15**.**7%**	**2**.**07**	**(1.37, 3.12)**	**0**.001*
ADHD + Stims	511	45	8.8%	0.953	(0.62, 1.46)	0.827	ADHD + Stims	511	57	11.2%	1.239	(0.83, 1.86)	0.301

## Results

**Figure 1:**
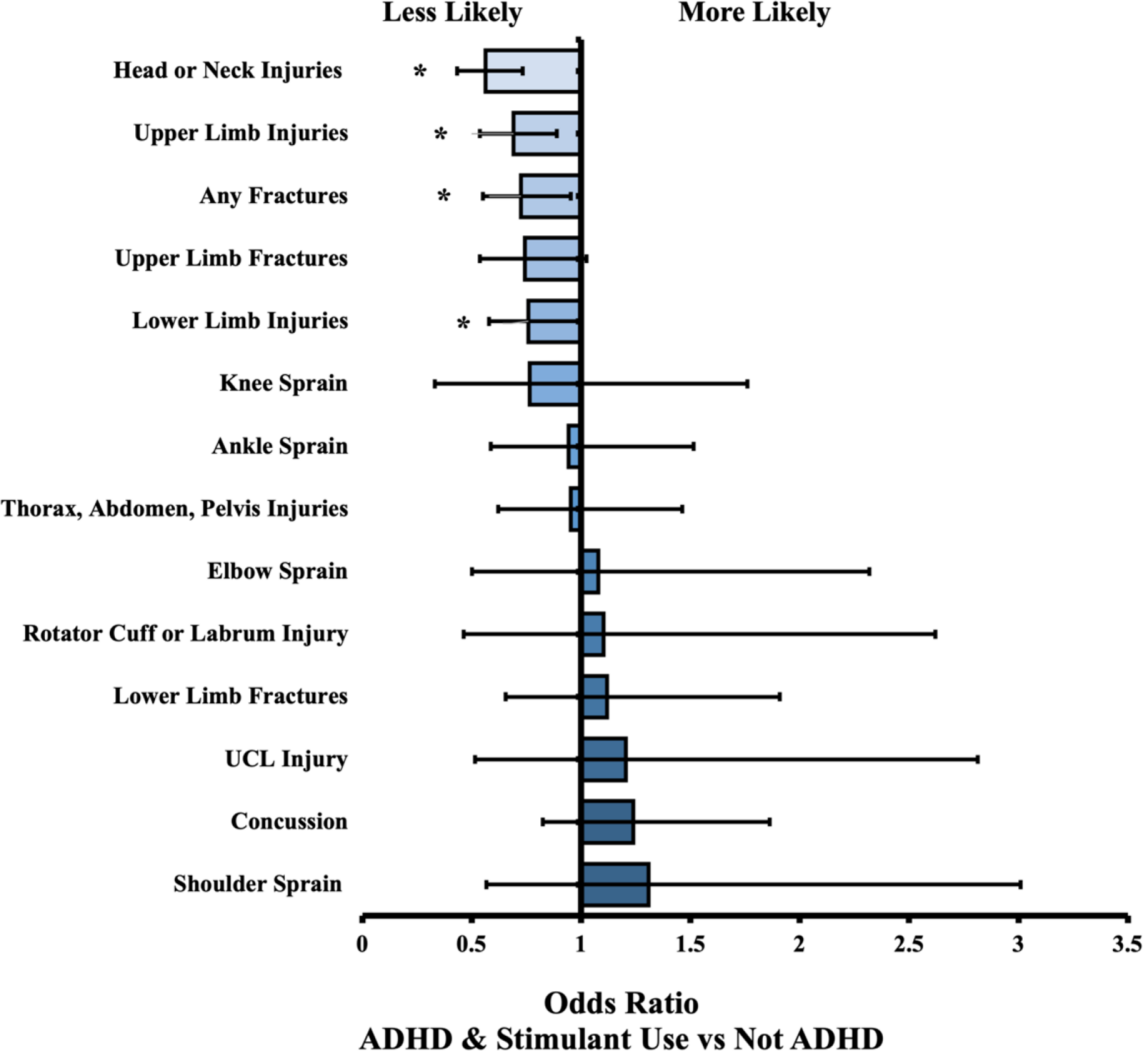
Odds ratio (OR) by injury type. OR < 1 indicates decreased likelihood of injury in ADHD (+) stimulant cohort compared to non-ADHD reference cohort. Confidence bars represent 95% interval. Asterisk (*) data labels indicate significant difference (*P* < 0.05).

**Figure 2:**
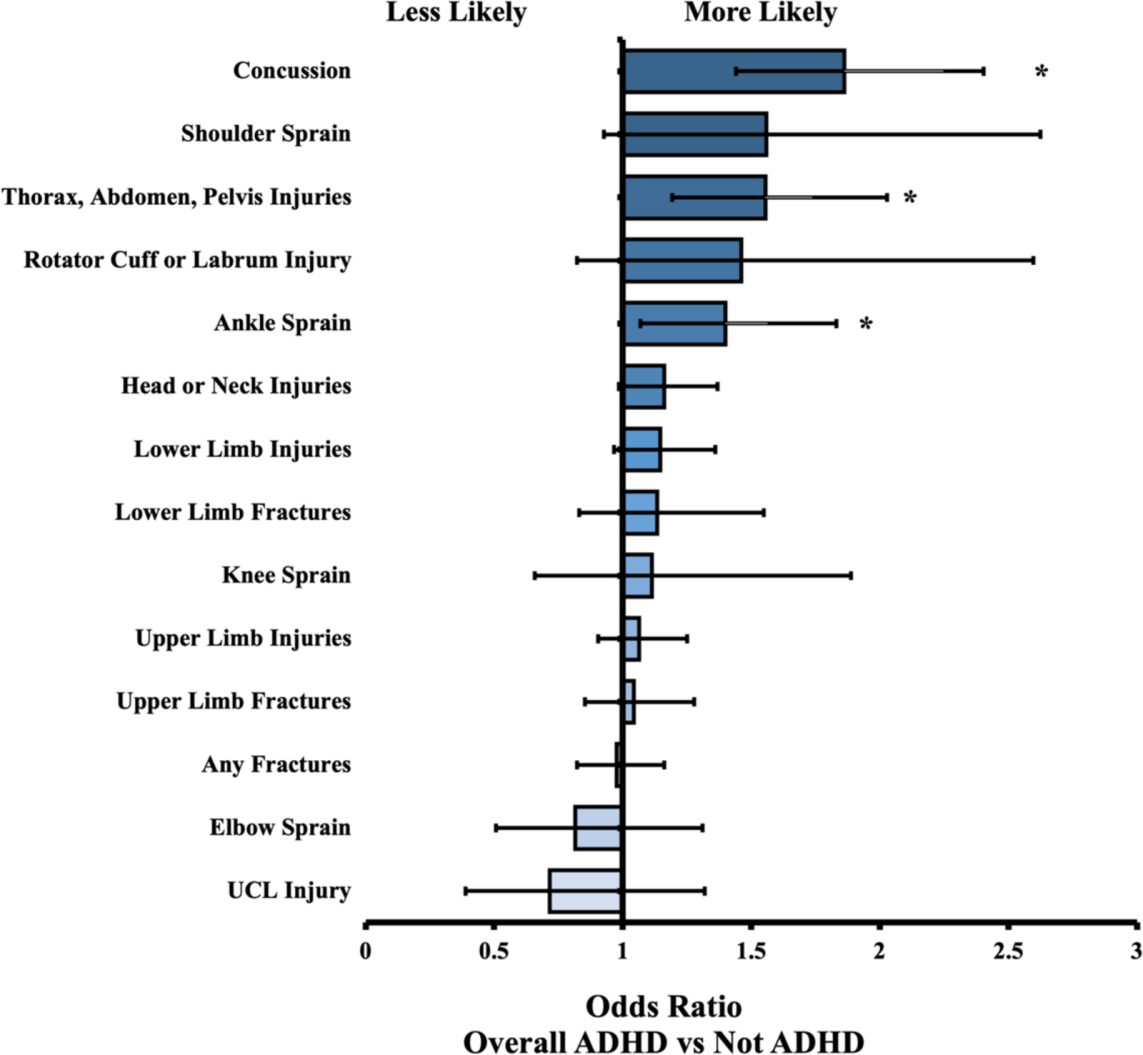
Odds ratio (OR) by injury type. OR < 1 indicates decreased likelihood of injury in Overall ADHD cohort compared to Not ADHD reference cohort. Confidence bars represent 95% interval. Asterisk (*) data labels indicate significant difference (*P* < 0.05).

**Figure 3:**
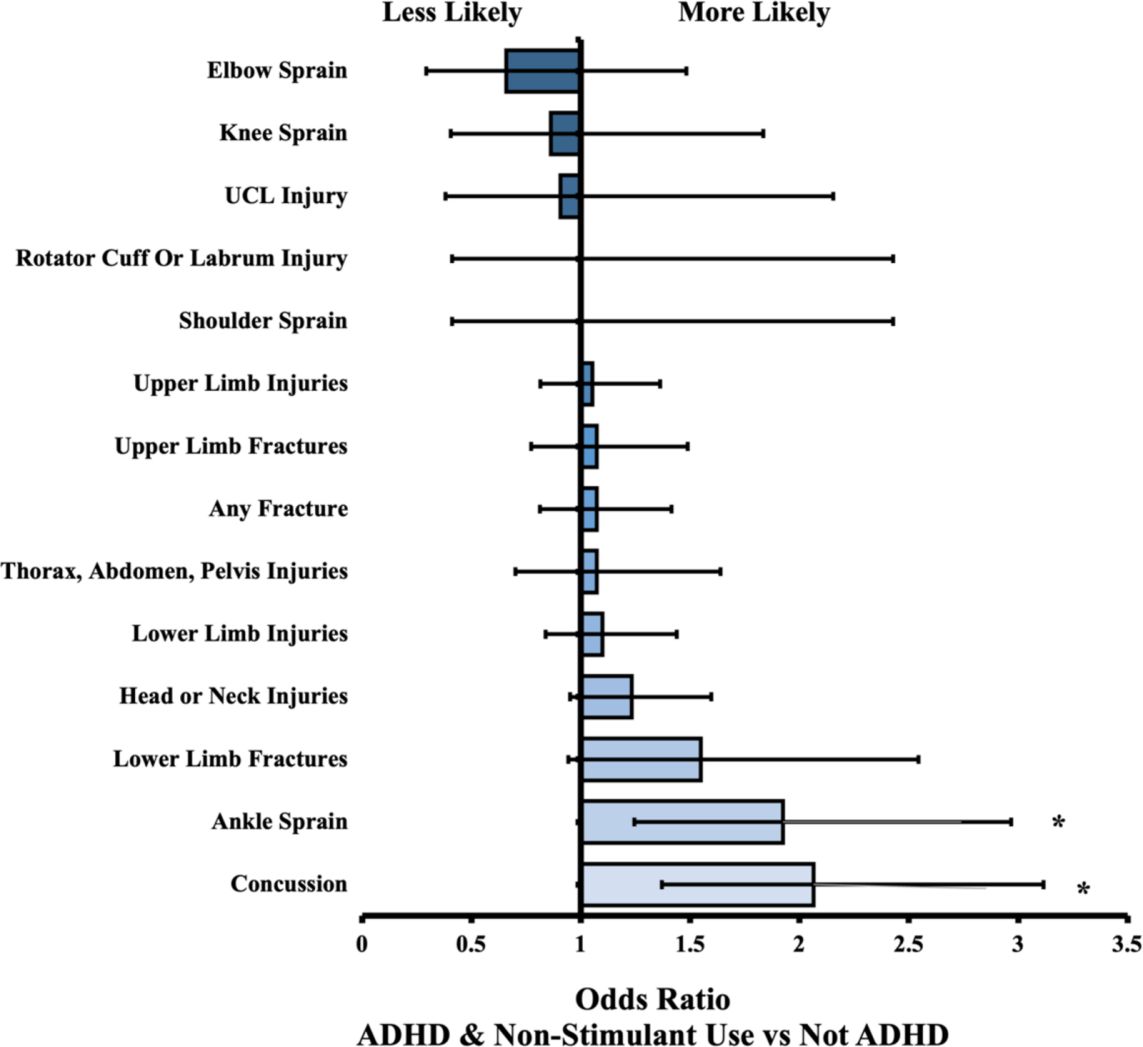
Odds ratio (OR) by injury type. OR < 1 indicates decreased likelihood of injury in ADHD non-stimulant cohort compared to Not ADHD reference cohort. Confidence bars represent 95% interval. Asterisk (*) data labels indicate significant difference (*P* < 0.05).

We identified 17,710 patients under 25 years old with designated baseball activity, 1,183 of which had a diagnosis of ADHD. Of these, 511 had a history of stimulant medication and 470 had no history of stimulant use. For most events (i.e., injuries), there were no statistical differences between cohorts. The overall ADHD cohort significantly differed from the Not ADHD cohort in 3 events: “thorax, abdomen, pelvis injuries” (OR = 1.56, *P* = 0.001), “ankle sprain” (OR = 1.40, *P* = 0.014), and “concussion” (OR = 1.86, *P* = 0.001). The no stimulant group differed in “ankle sprain” (OR = 1.92, *P* = 0.003), and “concussion” (OR = 2.07, *P* = 0.001). The stimulant group differed in 4 of the 14 measured outcomes but not in the 3 outcomes mentioned previously by other groups. Further results are in Table 2, along with odds ratios and confidence intervals. All relationships described were comparing the Not ADHD cohort to the relevant ADHD cohort. Therefore, an odds ratio above 1 indicates the ADHD cohort was more likely to experience the outcome of interest, and an odds ratio less than 1 indicates the ADHD cohort was less likely to experience the outcome. Figures 1, 2 present visual depictions of odds ratios between athletes without ADHD and patients with stimulant use (Figure 1) and overall ADHD (Figure 2). Figure 3 presents visual depictions of odds ratios between athletes without ADHD and patients with non-stimulant use.

## Discussion

Our findings present an interesting mix of injury differences across baseball athletes based on ADHD status and medication management. The comparison between patients without ADHD and the overall ADHD group revealed three injuries that are more common: “thorax, abdomen, pelvis injuries,” “ankle sprain,” and “concussion.” Within the category of “thorax, abdomen, and pelvis injuries,” the most common injury type is a fracture of the lumbosacral spine and pelvis (S32.9), which most often occurs in high-energy collisions (14), but there is also a specific type of pelvic avulsion fracture almost exclusively encountered in adolescent athletes, commonly in baseball (15). Ankle sprains are also very common in baseball, due to the combination of rapid acceleration, rotational force, and sliding maneuvers employed (8). The increased rate of sprains in patients with overall ADHD and ADHD without stimulant use could be caused by a variety of ADHD factors. For example, an athlete with decreased situational awareness may decide to slide without appropriate recognition of another player's positioning, leading to collision (16).

Concussion is an important event that is increased in both overall ADHD and ADHD without stimulant use. Research into associations between concussion and athletes with ADHD has been performed at all levels of baseball, including high school, college, and professional play. In one study, more than 50% of athletes with ADHD in NCAA Division I sports reported at least one prior concussion, compared to 14% of athletes without ADHD (17). Given the substantial negative impact of concussions on neurocognitive function, preventing concussions whenever possible is an important goal (17). Our data is in line with prior research, both for overall ADHD, ADHD without stimulant use, and the comparatively lower risk for concussion in athletes receiving stimulant medications (18, 19). Our cohort with ADHD and stimulant use showed no statistical difference in concussion occurrence compared to athletes without ADHD, which is further evidence in support of stimulant use preventing concussions in this population (19). Prior research has shown fractures involving the skull are less common in patients with ADHD on stimulants and that the detrimental effect of concussions that do occur is minimized in patients on stimulants compared to ADHD patients without stimulant use (18–20).

Concussions were not the only improved outcome present in our stimulant group. Compared to baseball players without ADHD, athletes with ADHD on stimulants were less likely to have fractures of the axial skeleton, less likely to have any fractures at all, and less likely to injure the head, neck, upper limb, and lower limb. Reduced fracture rates in ADHD patients on stimulants has been reported in prior research (20), but the decreased rate of baseball specific injuries in the limbs and head or neck is a significant finding with many possible explanations as to an underlying mechanism. For example, the decreased sensation of pain associated with chronic stimulant use may result in a decrease rate of reported injuries (21), even if the true occurrence rate were identical. It is also possible that the increased situational awareness, acceleration capability, and reaction speed associated with stimulant use allows these players to avoid collisions or mistakes that would have led to injuries in neurotypical players and ADHD athletes without stimulants (22). Without specific case-by-case analysis of the mechanism of injury, it is difficult to make definitive statements on the cause behind this strong association. However, the difference in risk is substantial enough to warrant further investigation.

Stimulant use is a controversial topic within the baseball community (23). Stimulants have many beneficial effects for youths with ADHD that go beyond academic performance (24), yet there is a persistent suspicion that some baseball players may be feigning ADHD symptoms in order to gain an exemption for stimulant use (25). This perception has led to large organizations like the MLB reducing the number of exemptions allowed over time, despite an increase in the overall prevalence of ADHD (26). Given the potential financial incentives of performance enhancing drug use in baseball, it is understandable that fans may be concerned regarding a potential advantage given to some players. However, our data indicate that stimulant use is associated with fewer head and neck injuries for adolescent players with ADHD. Head and neck injuries correspond to a significant amount of healthcare costs and lost playing time (22). While it is beyond the scope of this study, this difference in injury risk associated with stimulant use should be given considerable weight in discussions around medication regulations in sports.

As the average age in our study is 17.7 years old, our findings are more relevant for athletes at the high school and early collegiate level. Stimulants are the mainstay of ADHD treatment due to their highly beneficial effects on academic performance, as well as social development, emotional regulation, and overall mental health (13, 24). Stimulants are also associated with lower rates of injuries such as fractures in all patients with ADHD, not only baseball players (20). Therefore, it is crucial that individuals with ADHD do not experience undue restriction on ADHD treatment. While athletic scholarships may provide some incentive to feign ADHD symptoms to obtain stimulants, the fact remains that roughly 10% of youths in the United States have ADHD (1). Detecting and diagnosing true ADHD should be the responsibility of physicians and psychologists, and those with diagnoses should be able to continue using stimulant medications without stigmatization or undue restriction by athletic associations.

While our study avoids many potential confounders, such as age, sex, and race, there are some limitations that restrict the generalizability of our findings. The data obtained from TriNetX are deidentified and presented in aggregate form, which prevents analysis on an individual level and restricts important information such as severity of ADHD symptoms. Furthermore, electronic medical records are limited to information entered by health care providers; it is extremely likely that our findings are an underestimate of baseball injuries as we only included patients identified as playing baseball through their health record. Similarly, medications may be entered in the record but not taken. However, the large size of our study mitigates most of these limitations, and ensures our findings have some generalizability to ADHD athletes in baseball.

Given the growing population of ADHD youth, and the ongoing debate around stimulant use in athletics, our study is relevant to a wide variety of patients, providers, and the baseball community at large. Still, further research is needed to investigate potential causes behind the associations identified. As more is discovered about the link between ADHD and injuries, the use of stimulants in baseball should be continually reassessed. With collaborative effort, the rate of injuries in ADHD athletes might be reduced, allowing more players to continue enjoying the quintessential American pastime.

## Data Availability

The original contributions presented in the study are included in the article/Supplementary Material, further inquiries can be directed to the corresponding author/s.
